# Biomaterials Meet Microfluidics: From Synthesis Technologies to Biological Applications

**DOI:** 10.3390/mi8080255

**Published:** 2017-08-19

**Authors:** Jingyun Ma, Yachen Wang, Jing Liu

**Affiliations:** 1 Regenerative Medicine Center, the First Affiliated Hospital of Dalian Medical University, Dalian 116011, China; majingyun@dmu.edu.cn (J.M.); wangyachen100@163.com (Y.W.); 2 Stem Cell Clinical Research Center, the First Affiliated Hospital of Dalian Medical University, Dalian 116011, China

**Keywords:** functional biomaterials, microfluidics, controllable synthesis, biological applications

## Abstract

Microfluidics is characterized by laminar flow at micro-scale dimension, high surface to volume ratio, and markedly improved heat/mass transfer. In addition, together with advantages of large-scale integration and flexible manipulation, microfluidic technology has been rapidly developed as one of the most important platforms in the field of functional biomaterial synthesis. Compared to biomaterials assisted by conventional strategies, functional biomaterials synthesized by microfluidics are with superior properties and performances, due to their controllable morphology and composition, which have shown great advantages and potential in the field of biomedicine, biosensing, and tissue engineering. Take the significance of microfluidic engineered biomaterials into consideration; this review highlights the microfluidic synthesis technologies and biomedical applications of materials. We divide microfluidic based biomaterials into four kinds. According to the material dimensionality, it includes: 0D (particulate materials), 1D (fibrous materials), 2D (sheet materials), and 3D (construct forms of materials). In particular, micro/nano-particles and micro/nano-fibers are introduced respectively. This classification standard could include all of the microfluidic biomaterials, and we envision introducing a comprehensive and overall evaluation and presentation of microfluidic based biomaterials and their applications.

## 1. Introduction

Since the 1990s, no matter in the field of natural science, nor engineering technology, miniaturization has become one of the general development trends [1,2,3]. A microfluidic system, namely, lab-on-a-chip, is a multifunctional platform which integrates basic operating units involved in the fields of chemistry and biology, such as sample preparation, reaction, separation, detection, and cell culture, separation, lysis, into a chip, within an area of a few square centimeters [4,5]. In this system, the micro-structured units and controllable fluidics constitute the network, which work as conventional chemical or biological laboratories [6,7]. The idea of microfluidics fits well with the concept of miniaturization, and thanks to its interdisciplinary advantages, it has been widely applied in fields such as engineering, physics, chemistry, microscopy, and biotechnology [8,9,10].

Biomaterials refer to a class of materials that have specific biological functions. Generally, biomaterials include any substance engineered to be a part of the biological system, or play a template role which could be sacrificed, either for a therapeutic purpose, or a diagnostic one [11,12]. Biomaterials science is a subject of cross disciplines, including medicine, biology, and chemistry [13,14]. With the progress of biomaterials science, considerable research has focused on micro/nanometer-scale materials with complex structures, because the microscopic architectures enable biomaterials great optimized properties [15]. Conventional bulk synthesis usually adopts the certain physical or chemical method (e.g., mechanical stirring) [16,17]. These methods generally generate products with monotonous morphology, and the dispersity of the biomaterials and production process are difficult to control. Although there have been efforts to improve the homogeneity of products, such as DeSimone’s print technique [18,19] and membrane emulsification [20], the lack of manipulation flexibility still limits the improvement of materials synthesis. In particular, for biomaterials that are specific, such as “intelligent biomaterials” or “functional biomaterials”, the conventional approaches are insufficient to meet their synthetic requirements. Therefore, it is extremely urgent to develop novel synthesis technologies for functional biomaterials. At this point, characterized by the microscale and rapid process, microfluidics, comes into sight and arouses the great interest of researchers.

Micro/nanometer-scale functional biomaterials synthesized from the microfluidic platform are of different configurations, complex structures, and novel properties in flexible and easily prepared way [21,22]. Compared to conventional synthetic methods, the advantages of microfluidic synthesis lie in following aspects. The biomaterial size, morphology, and composition are easily controlled, resulting in superior properties. In the micro-scale system, the reaction could be accelerated due to low consumption of reagents, rapid heat transfer, and mass transfer. The internal reaction conditions are stable, with no cross contamination. The preparation is of spatiotemporal resolution, and with controllable addition of the reagent, which is beneficial to the process of multi-step and multi-reagent synthesis. Through the scale integration of a microfluidic system, flexible manipulation, and equip automation, the complex reaction process can be simplified. Due to the minute volume of the microfluidic chips, and match of most chips’ materials to the microscopic observations, real-time monitoring of the reaction process in the chip could be realized, which helps to clarify the synthesis mechanism [23,24]. Therefore, the use of microfluidic technology to design and prepare functional biomaterials has become a hot topic recently, moreover, will continue to bring infinite possibilities for the future development of the areas of materials science and biology [25,26]. 

**Figure 1:**
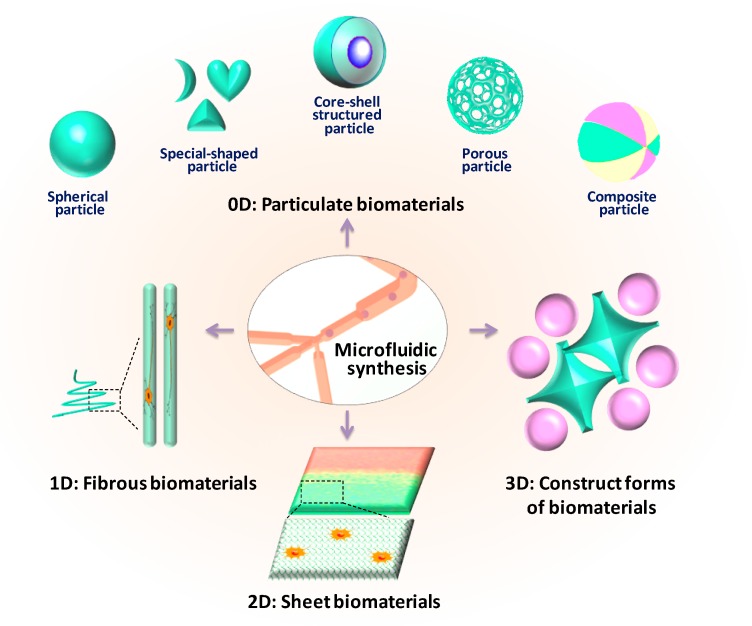
Schematic diagram of the classification of biomaterials engineered from microfluidics.

To date, there have been several excellent reviews on the microfluidic fabrication of materials, from different points of view [27,28,29], which mainly deal with certain microfluidic technologies (e.g., droplet microfluidic) or specific forms of materials (e.g., Janus particles). A comprehensive review pinpointing the microfluidic synthesis and bio-applications of materials is still lacking. In this review, we summarize the microfluidic methods for preparation and applications of all kinds of biomaterials, both at micro-scale and nano-scale, and in definite classification, from 0D to 3D forms. Herein, classical and recent achievements in the biomaterials engineered from microfluidics are presented and categorized. According to the material dimension, namely, dot, line, face, and body, microfluidic biomaterials are classified into four main categories, that include zero-dimensional particulate materials (0D), one-dimensional fibrous materials (1D), two-dimensional sheet materials (2D), and three-dimensional construct forms of materials (3D), as shown in Figure 1. In particular, micro/nano-particles and micro/nano-fibers are introduced respectively. More specifically, 0D materials refer to that with sizes in every direction at the same order of magnitude, presenting “particle” appearance. For micro/nano-particles, the sizes in every direction are all at micro/nano scale, respectively. For 1D material, size in one direction is several orders greater than those in other directions, presenting “fiber” appearance. For micro/nano-fibers, the obvious size in certain direction maybe at millimeter even meter-scale, while the sizes of other directions are micro/nano scale, respectively. For 2D material, size in one direction is several orders less than those in other directions, presenting “sheet” appearance. Similar to 0D materials, 3D materials refer to that with sizes in every direction at almost the same order of magnitude, presenting “construct” appearance. However, the sizes of 3D materials in at least two directions are at millimeter scale. Compared to 2D material, 3D materials are more stereoscopic.

## 2. Particulate Biomaterials Synthesis and Applications

Traditional methods of preparing particulate materials include mechanical agitation, emulsion polymerization, seeding polymerization, precipitation, etc., however, due to the broad particle size distribution and uncontrollable morphology, their applications are greatly restricted. Because of the advantages of uniform particle size and controllable morphology, particulate biomaterials synthesis from microfluidics has become a hot topic in recent years [30,31]. In general, microfluidic technologies for particulate biomaterials synthesis are composed of droplet microfluidics [32] and photolithography [33].

**Figure 2:**
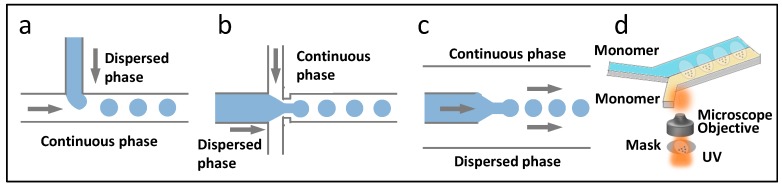
Microfluidic technologies for the synthesis of particulate materials. The principles and chip designs with different flow regimes for droplet generation, including T-junction (**a**); flow-focusing (**b**); and coaxial (**c**) structured chip; (**d**) Photolithography applied in microfluidic synthesis.

Droplet microfluidics uses fluidic manipulation at the micro scale, dispersing one liquid into another liquid that is immiscible, generating independent liquid units in the microfluidic channels [34]. Correspondingly, a multi-spatial reaction synthesis system is constructed, which is located on the inside of the liquid drop, the liquid drop edge, and the liquid drop interval [35]. In droplet microfluidics synthesis, the dimensions, shape, and monodispersity of the droplets, can be precisely controlled, and enable the method one of the most commonly used microfluidic material synthesis means. The principles and chip designs for droplet generation are as shown in Figure 2, including T-junction (Figure 2a), flow-focusing (Figure 2b), and coaxial (Figure 2c) structured chip. In T-junction chip, the dispersed phase flows from a vertical channel to a horizontal channel, filled with the continuous phase. Under the combined action of shear force and extrusion pressure both from continuous phase, monodispersed droplets are generated. In the flow-focusing chip, the dispersed phase flows from the middle channel, and suffers extrusion force of the continuous phase from both sides. The dispersed phase experiences stretching and breakage, leading to droplet formation. While for the coaxial structured chip, the dispersed phase channel is embedded in the continuous phase channel, and the dispersed phase flows parallel to the continuous phase towards the same direction. As well, the dispersed phase is broken into droplets. In the microchannels, droplet generation is influenced by the microchannel construction, viscosity and flow velocity of the two phases, and interfacial tension between each adjacent phase. Therefore, the dimension and production rate of the droplets can be controlled by adjusting the above parameters. In addition, double or even multiple emulsions could be generated for particulate material synthesis with core-shell or multi-core structure, through the composite design of microchannels. 

Particles from droplet microfluidics are mainly limited to spherical or simple spherical variations, such as hemisphere, cylindrical or pie-like. Besides, in some cases, a chemical modification is needed to guarantee a continuous and smooth formation and flow of the droplets in the microchannel. In order to improve the limitations of droplet microfluidics, photolithography is put forward to microfluidic synthesis, which combines microfluidics and photolithography [36], as shown in Figure 2d. In this method, monomer solution that is sensitive to ultraviolet light is introduced into the single channel, and one side of the chip is covered by the mask that contains the design pattern. After ultraviolet irradiation, partial polymerization of the monomer solution endows the materials with specific shapes. According to the monomer polymerization rates, the fluid can be applied with lights in continuous flow or flow–stop–flow manner. Although photolithography enriches the material shape of microfluidic synthesis, it is only suitable for light-sensitive materials.

The specific and primary shape could be given to the biomaterials by microfluidic technology, however, regardless of the particulate, fibrous, sheet, or construct forms of materials, specific solidification methods are needed to get the final form of the material. Principles and methods of materials solidification involved in microfluidic synthesis include polymerization, solvent volatilization and solvent extraction, ionic crosslinking and chemical crosslinking for organic materials, and inorganic chemical reaction and self-assembly for inorganic materials. 

The polymerization reaction is the method for preparing polymer functional materials using monomer as the raw material. The monomer solution in the microchannel is as the dispersed phase or continuous phase, and solidified by polymerization into a particular shape. According to the reaction triggering modes, the polymerization method can be divided into light polymerization, thermal polymerization and polymerization for which only the initiator is needed, without external light or heat stimulation. In light polymerization, the monomer solution is exposed to the light and polymerized in the channel, and the materials are collected from the channel after basic curing. Heat polymerization is triggered by heating the polymer system, or by simply reducing the temperature of the solution with high melting point. The initiator polymerization needs the addition of a certain chemical that triggers the polymerization process. Crosslinking reactions, such as ionic crosslinking and chemical crosslinking, are formed between the polymer chains and crosslinkers through covalent or noncovalent bonds. Solvent evaporation and solvent extraction both utilize the polymer as the raw material. In the solvent evaporation method, the volatile solvent evaporates from the polymer solution, and the polymer is solidified, while for solvent extraction, the solvent is extracted by another chemical, and the polymer is separated out and solidified. For microfluidic inorganic materials synthesis, the raw materials experience various inorganic reactions in the microchannels, such as hydrolysis, reduction, precipitation, etc. In particular, based on this reaction principle, a variety of nano-functional biomaterials could be synthesized in the microfluidic device. Self-assembly refers to the particles or molecules being self-assembled, forming materials with more complicated construction and multidimensional performance.

Accompanied by the development of technologies and theories as mentioned above, microfluidics-based particulate biomaterials synthesis and applications have experienced a process, from simple to complex. A series of particulate biomaterials, such as spherical particles, special shape particles, porous particles, core-shell structured particles, and even multi-component composite particles, have been developed. 

### 2.1. Particulate Biomaterials at Micro-Scale

#### 2.1.1. Spherical Microparticles

**Figure 3:**
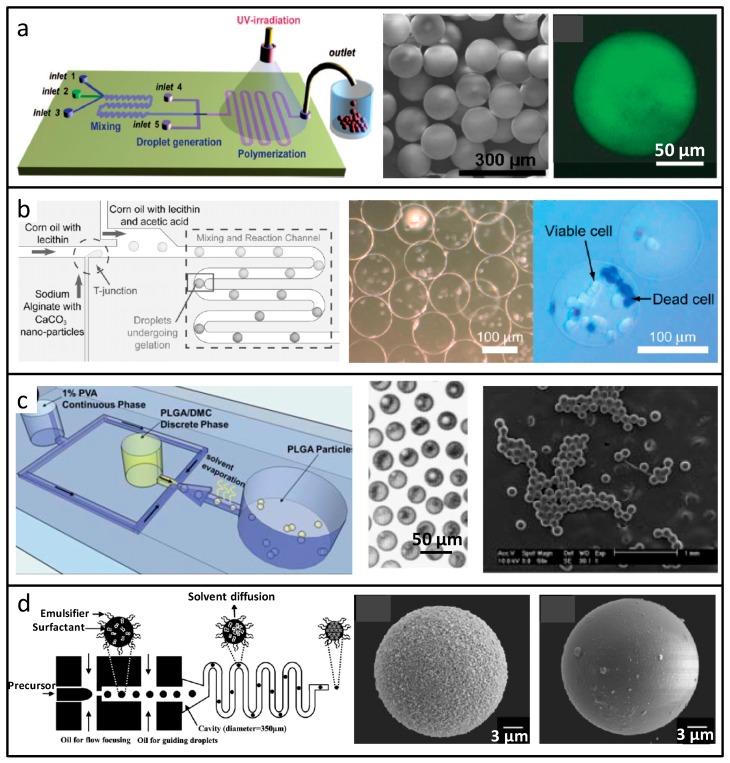
Spherical microparticles prepared by droplet-based microfluidics. (**a**) Microspheres from ultraviolet (UV) or heat-induced polymerization in microfluidic droplets; (**b**) Calcium alginate microbeads for cell encapsulation; (**c**) Oil-in-water (O/W) droplet synthesis of poly lactic-co-glycolic acid (PLGA) microspheres; (**d**) SiO_2_ microspheres generated based on sol-gel principle and microfluidic droplets. Reproduced with permission from [38,40,41,44].

In the absence of any external force, droplets tend to be spherical, to keep the minimum surface energy and most stable condition, therefore, the synthesis of micro-spherical materials had been first realized in the microfluidic system. In 2000, Nakajima and co-workers reported the first research work on microsphere synthesis in the microfluidic device [37]. Based on the thermal polymerization principle, firstly, at high temperatures, oil-in-water (O/W) droplets were generated. After the processing of curing and freeze-drying, the lipid microspheres were with an average dimension of 20 μm, and variable coefficient less than 5%, which improved the particle size distribution effectively, compared to conventional suspension polymerization suitable for microsphere synthesis above 10 μm. Kumacheva and co-workers dispersed the polymer monomer into droplets on the microfluidic chip. The droplets were turned into microspheres by ultraviolet light or heat-induced polymerization, as shown in Figure 3a [38]; the droplets could be generated steadily, received the external stimulus in turn, and transformed into polymer microspheres with a uniform particle size in a single dispersed form. This microsphere synthesis is stable and simple, but only suitable for photosensitive or heat-sensitive raw materials. Shibata and co-workers introduced a fluorescent dye with glucose responsiveness into the monomer, and this functional monomer was emulsified and polymerized in the chip to form gel microspheres [39]. The fluorescence intensity of microspheres injected into the mice represented different levels of glucose in the body, and the real-time monitoring of glucose concentration was achieved. 

The preparation of biocompatible materials, which can be used for the drug release, cell cultivation, and protein purification, has become one of the research hotspots. Among them, biocompatible calcium alginate and chitosan materials are usually prepared based on crosslinking, namely, the polymer solution is dispersed as an emulsion in the microchannel, and the crosslinking agent is then introduced, and triggers the crosslinking reaction that allows the emulsions to solidify. Takeuchi and co-workers prepared uniform calcium alginate microbeads according to the principle of internal gelation, which were used for leukemia cell encapsulation [40]. As shown in Figure 3b, in the “T” structure of chip, the sodium alginate was dispersed into droplets with cells and CaCO_3_ nanoparticles. By adding continuous phase containing acetic acid in the downstream channel, CaCO_3_ nanoparticles inside the droplets released the Ca^2+^, and triggered the gelation reaction to form the microcapsules. 

Microspheres prepared by the O/W liquid drop synthesis system have extensive applications in the field of drug release. In general, biocompatible and biodegradable polymer materials, such as poly lactic-co-glycolic acid (PLGA), polycaprolactone (PCL) and polylactic acid (PLA), are dissolved in an organic reagent as the dispersed phase. After emulsion and solvent evaporation, the microspheres are generated [41,42,43]. The microsphere particle size is determined by the size of the droplets, and the concentration of the polymer. Take PLGA microspheres, for example, as shown in Figure 3c. Firstly, PLGAwas dissolved in dimethylcarbonate, as the dispersed phase. At the flow-focusing structure of chip, this fluid was emulsified into droplets by the continuous phase containing 1% polyvinyl alcohol (PVA) as the surfactant. After complete volatilization of the solvent dimethylcarbonate, PLGA microspheres were solidified. 

Utilizing the hydrolysis reaction of the inorganic oxide precursor, sol solution was generated and emulsified into droplets, and after solvent dissipating, inorganic salt oxide microspheres were synthesized; a typical example is the synthesis of SiO_2_ microspheres [44], as shown in Figure 3d. It is expected that these ordered mesoporous silica microspheres might be useful for biomolecule delivery.

#### 2.1.2. Special-Shaped Microparticles

The acquisition of the spherical shape of the solid microspheres only relies on the most steady state of the droplets, and the synthesis is relatively simple and reflects research in the early exploration stage of microfluidic material synthesis. Accompanied by the development of microfluidic technology and growing demand of advanced biomaterials, researchers shifted their gaze to the preparation of special-shaped microparticles, such as the hemisphere, round cake, crescent, polygon, and stick. Besides uniform material size, versatile shape and morphology of materials are the more prominent advantages of microfluidic synthesis. 

**Figure 4:**
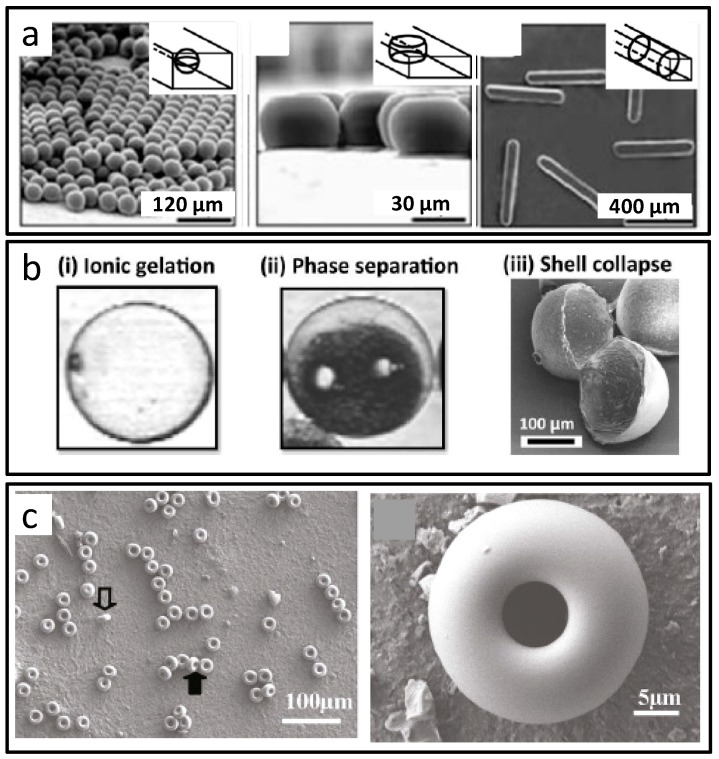
Special-shaped microparticles prepared by droplet-based microfluidics. (**a**) Poly tripropyleneglycol diacrylate particles with different shapes determined by the microchannel dimension; (**b**) Polylactic acid (PLA) crescent-shaped composite microparticles from core-shell microcapsules in the microfluidic chip; (**c**) One-step prepared doughnut-shaped SiO_2_ microparticles. Reproduced with permission from [45,47,48].

By the combination of the droplet microfluidic technology and microchannel configuration design, deformation of the droplet was realized. After in situ light polymerization or refrigeration, non-spherical solid particles were achieved [45]. As shown in Figure 4a, when the droplets diameter was smaller or greater than both of the microchannel depth and width, the particles presented as spherical or short bar; when the droplets diameter was greater than the microchannel depth and smaller than the microchannel width, the particles presented as a round pie. The method illustrated visually that by adjustment of the droplet size and the microchannel size, special-shaped microparticles could be formed by the squeezing effect. Taking advantage of local surface modification of the chip channel and two droplet forming units, Zhang and co-workers obtained double emulsion droplets oil-in-water-in-oil (O/W/O). By changing the flow rates, the number of internal oil cores could be adjusted. Under the synergistic effect of geometric limitations of microchannels and inhibited interfacial polymerization, and by removing the internal oil cores, meniscus or multi-foot hydrogel microparticles were synthesized [46]. 

Vladisavljevic et al. developed core-shell microcapsules in the microfluidic chip, which could be turned into crescent-shaped composite microparticles after drying, due to the collapse of PLA shells encompassing water-rich crescent regions, as shown in Figure 4b [47]. These crescent-shaped microwells could be used for cell trapping/immobilisation. Doughnut shaped SiO_2_ microparticles were prepared in one-step by Douliez and co-workers. The reduction of solvent in silica gel caused a change in gelation, and the curvature instability at the droplet interface induced depression in the cross-flow direction, leading to the formation of a ring structure, as shown in Figure 4c [48].

**Figure 5:**
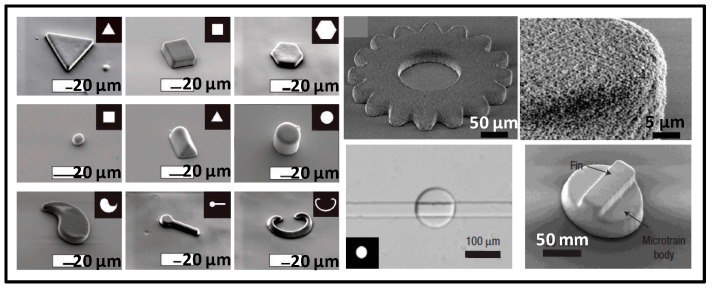
Special-shaped microparticles prepared by photolithography -based microfluidics. Reproduced with permission from [49,50,51].

Doyle and Kwon’s groups performed a series of works on the use of photolithography to prepare microparticles. As shown in Figure 5, a monomer solution that was sensitive to ultraviolet (UV) light was introduced into the channel, and one side of the chip was covered with the mask that contains the design pattern. After the UV exposure, the monomer solution was partially polymerized, due to the patterned masking effect. Using flow photolithography, special-shaped microparticles in triangles, circles, squares, polygons, and ring shapes could be obtained in the microfluidic chip [49]. Similarly, gear shaped polyacrylamide-silicon composite particles were generated [50]. Multilayer structured microparticles could be fabricated by the combination of photolithography and 3D design of the chip [51].

#### 2.1.3. Core-Shell Structural Microparticles

Microparticles with micro-hierarchical structure, such as core-shell and porous structure, are highly regarded due to their advanced properties. Firstly, the successful syntheses of these materials with special configurations reflect the improvement of modern synthetic techniques. Next, these materials facilitate further understanding of the mechanism of microstructure formation, thus encouraging inspiration into the design of more innovative materials. Once more, relative to the microparticles with simple structure, hierarchical structure endows materials with novel construction and superior mechanical, optical, and other properties. Finally, the excellent material performance will greatly enrich and develop practical application in the biology field. 

Core-shell microparticles refer to particles of which shell and kernel are made up of different substances. The shells are generally solid, and the kernel would be one or more of solid, liquid, and gaseous states. The whole materials consists of special structures like yolk–egg white, yolk–shell and empty shell. The core–shell microparticles realize the internal hierarchy of the material. The compartments could be with independent carrying capacity and function, and at the same time, the compartments could participate in the overall function exertion of the material in the synergetic manner.

**Figure 6:**
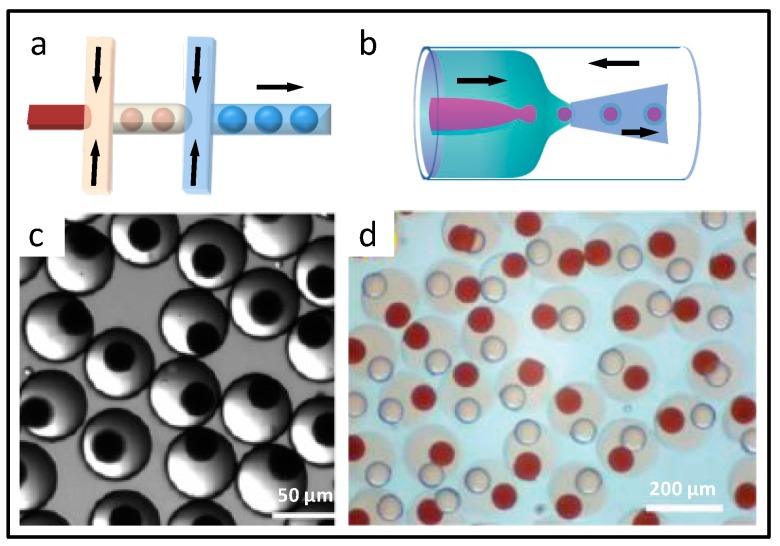
Core-shell structure microparticles prepared by multiple emulsion-based microfluidics. (**a**,**b**) Designs of microfluidic devices for the preparation of double emulsion droplets; (**c**) Microparticle synthesis with magnetic core and hydrogel shell from double-emulsion microfluidic droplets; (**d**) Microparticles with two independent oil cores wrapped in the hydrogel shell. Reproduced with permission from [53,54].

Synthesis of the core–shell microparticles commonly uses multi-emulsion droplets as the template. Figure 6a illustrates the schematic diagram of double emulsion droplet generation in the chip. Take O/W/O double emulsion droplets for example, in order to form oil in water droplets in the first emulsion unit, hydrophilic modification should be performed for the first emulsion unit, whilst hydrophobic modification should be performed for the second emulsion unit, to enable the intermediate water phase change from the continuous phase to the dispersed phase, for the generation and stable existence of the double emulsion [52]. As shown in Figure 6b, in a capillary microfluidic device with nested structures, the double emulsion could be one-step generation. Similarly, by adding the emulsion unit in the chip, the synthesis of multiple emulsion droplets can be realized. 

The O/W/O double emulsion droplets with hydrogel precursor shell and magnetic monomer core were used for microparticle synthesis [53]. As shown in Figure 6c, the shell part was photopolymerized, and the core was thermal polymerized. Under the external magnetic field, the microparticles could be manipulated via the magnetic core. Zhao et al. dispersed quantum dots (QDs) and magnetic nanoparticles into photochemical resin, respectively, and the two independent oil cores were wrapped in the same hydrogel shell [54], as shown in Figure 6d. These microparticles were with functions of transmitting optical information by the QDs cores, magnetic response characteristics by the magnetic nanoparticle cores, and biocompatibility by the hydrogel shells. It was effective to avoid contact with toxic materials in the internal matrix. Anderson et al. developed microfluidic microcapsules incorporating colloidal nanosensors, which could be used for biomolecular sensing [55]. The microcapsules with polyethylene glycol shell and liquid cores can selectively encapsulate nanosensors within the core, and guarantee free diffusion of molecules. Glucose and heparin were detected by glucose-responsive quantum dots/gold nanorods, and heparin-responsive gold nanorods, respectively.

**Figure 7:**
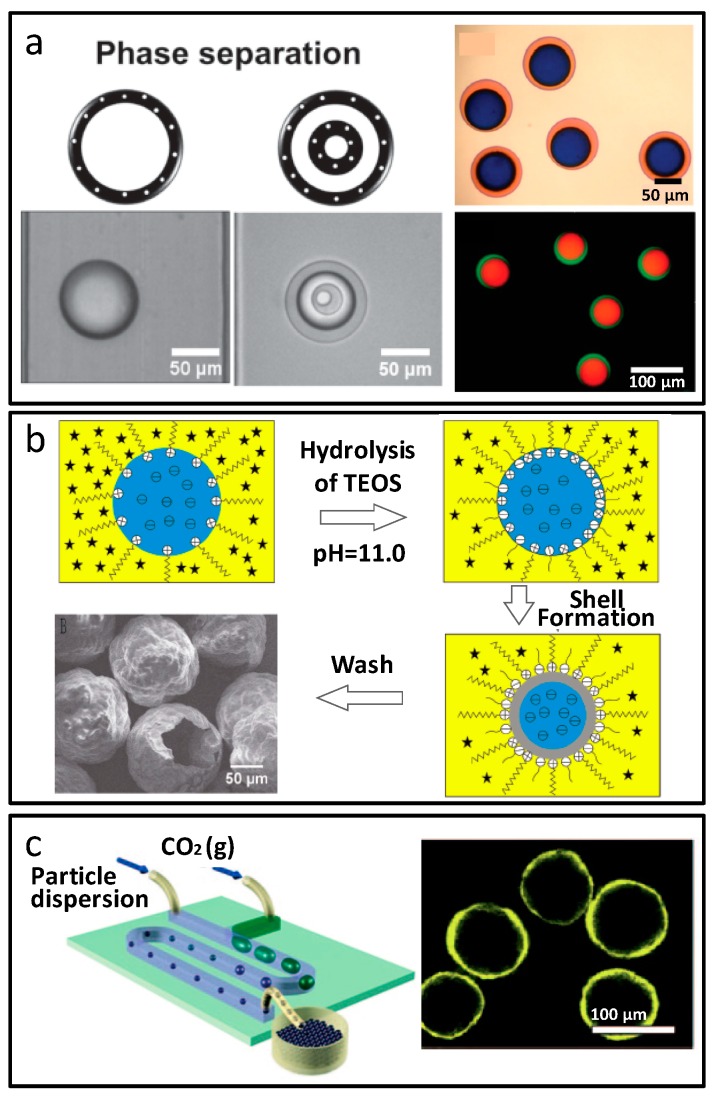
Core-shell structure microparticles prepared by phase separation and phase interface effect-based microfluidics. (**a**) Phase separation process of the polyethylene glycol diacrylate (PEGDA) core-shell microparticles; (**b**) Hollow SiO_2_ microspheres from interfacial reaction; (**c**) Interface self-assembly induced hollow structured microcapsules. Reproduced with permission from [56,58,59].

Core-shell structure microparticles could also be generated based on phase separation. W/O droplets were formed in the chip, and the separation reagents in the continuous phase spread into the polyethylene glycol diacrylate (PEGDA) water phase, forming the core-shell structure. The phase separation process was shown in Figure 7a [56]. By adjusting the concentration of PEGDA, multi-wall microspheres could be obtained, which was known as the “onion” structure. Zhang et al. prepared yolk–shell zeolitic imidazolate framework (ZIF)-8/alginate microcapsules using microfluidics [57]. Yolk–shell W/O ZIF-8 Pickering emulsions were the template of ZIF-8/alginate hybrid shell generation, and the yolks were from phase separation of polyethylene glycol and acrylamide solution induced by polymerization. Multi-yolk structures could be obtained by droplet coalescence. Rhodamine B controlled release of the materials showed drug carrier potential.

Based on the phase interface effect, such as the interface reaction and interface self-assembly, hollow microparticles could be synthesized, which is a kind of special, and the most simple, core–shell microparticle. The interfacial reaction refers to the reaction at the phase interface induced by the chemicals in the two phases, and presents a shell-like product. As the reaction progresses, the increased shell thickness inhibits the diffusion of reactant molecule across the two phases, and ends the reaction, leaving an empty shell structure. Typical work, such as hollow SiO_2_ microspheres synthesized by James Yang and co-workers [58], is shown in Figure 7b. Interface self-assembly is based on the self-assembly of colloidal particles or molecules at the two phase interfaces. The Kumacheva group used CO_2_ as the dispersed phase, and colloidal particle suspension as the continuous phase, to form uniform bubbles in the chip. As CO_2_ dissolved in the continuous water phase, the bubble volume and water phase pH decreased, and led to the assembly of colloidal particles at the gas–liquid phase interface. Microcapsules with a hollow structure were formed [59], as shown in Figure 7c.

#### 2.1.4. Porous Microparticles

Porous microparticles can be classified into microporous, mesoporous, and macroporous structures, with the corresponding pore sizes of lower than 2 nm, from 2 to 50 nm, and greater than 50 nm, respectively [60,61]. The pores can only exist on the surface of the material and can also penetrate the entire particle. Compared with the solid materials, such a porous structure greatly increases the specific area and light weight. 

**Figure 8:**
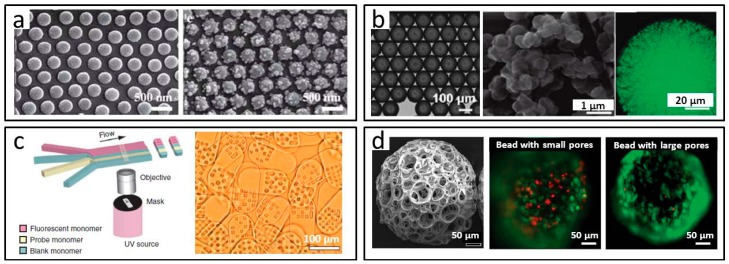
Porous microparticles prepared by microfluidics. (**a**) Hierarchically-structured microsphere surface with decorated particles; (**b**) Microparticles prepared with a network from the surface to the inside from microfluidic O/W droplets; (**c**) Flow lithography based encoded microparticles; (**d**) Cell carrier microparticles with controllable pore sizes. Reproduced with permission from [62,63,64,65,67].

Microspheres with a porous surface can be formed by template occupying and removing, or modified with small size particles on the particle surface. Yang and co-workers fabricated microspheres with hierarchical surface nanopatterns in a microfluidic system [62]. As shown in Figure 8a, the silica particles in photocurable droplets protruded across the interface as hexagonal arrays, and after polymerization, the silica particles were selectively decorated with silver nanoparticles, which could be used as hot spots for molecular detection based on surface-enhanced Raman scattering (SERS). Further functions of the microparticles were explored for structural colored or magnetoresponsive microspheres. In the work of Kumacheva et al. [63], the dispersed phase was a homogeneous mixture of monomers and solvents, and emulsified as O/W droplets. As shown in Figure 8b, after phase separation induced by photopolymerization and porogen solvent removing, uniform microparticles were prepared with a network from the surface to the inside. The great specific surface area of the materials showed their application prospects in the biomedical field.

As shown in Figure 8c, by using flow lithography approach, Doyle et al. combined particle fabrication, encoding, and probe incorporation into a single step [64,65]. The microparticles had one part with a unique square macroporous pattern, while the other part contained a probe molecule with a unique signature, which could be used for scanning DNA and proteins in a single sample. It was expected to promote the development of low-cost clinical diagnosis. 

Qin and co-workers synthesized biomimetic honeycomb microparticles based on the synergistic effect of double emulsion templates, polymer precipitation, and effervescent salt decomposition in the microfluidic chip [66]. The materials were composed of an external nanoporous membrane and internal cavities, and the biological functions as microcarriers in drug delivery and cell culture were explored. In the work of Xia et al., as shown in Figure 8d [67], porous microparticles with several micron holes, or dozens of micron holes, were prepared. Different separated layers of the W/O initial emulsion were selected for W/O/W double emulsion generation in the capillary coaxial structure. After organic solvent volatilization and water phase removal, through-pore microparticles with different pore sizes were achieved. Successful inoculation and continuous cultivation of fibroblast indicated a better biocompatibility of the PLGA microspheres with macropores.

#### 2.1.5. Composite Microparticles

Composite microparticles are a kind of material with different compositions, and functional characteristics beyond single component particles. Although there is some overlap with the special shaped or structured materials, in consideration that multicomponent would play a decisive role in the function of materials under some situations, composite microparticles are listed as a separate category.

**Figure 9:**
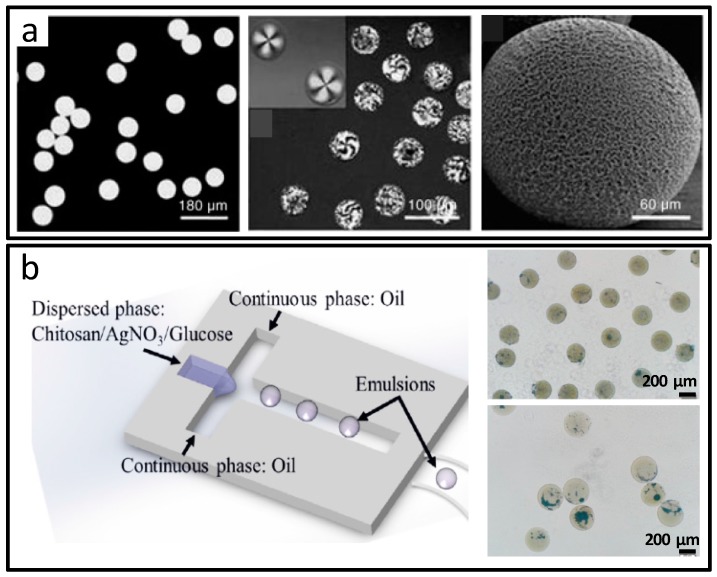
Composite microparticles prepared by microfluidics. (**a**) Microspheres labeled with 4-amino-7-nitrobenzo-2-oxa-1,3-diazole (NBD) dye and CdSe quantum dots; (**b**) Silver nanoparticle–chitosan composite microparticles assisted by microfluidic synthesis. Reproduced with permission from [45,68].

The most straightforward composite materials could be generated by adding different kinds of materials to the dispersion phase, such as dyes, nanoparticles, quantum dots and biomolecules. By polymerization or other reactions, the composite materials are formed with various functions. Whitesides et al. blended fluorescent dyes, CdSe QDs, pore-foaming agents, magnetic particles and liquid crystals into the tripropylene glycol diacrylate (TPGDA) photopolymer monomer solution for fabrication of the composite microparticles, as shown in Figure 9a [45], which displayed the diversity and simplicity of additive component manipulation for material synthesis. Silver nanoparticle loaded chitosan composite microparticles used for antibacterial were also reported, as shown in Figure 9b [68]. 

**Figure 10:**
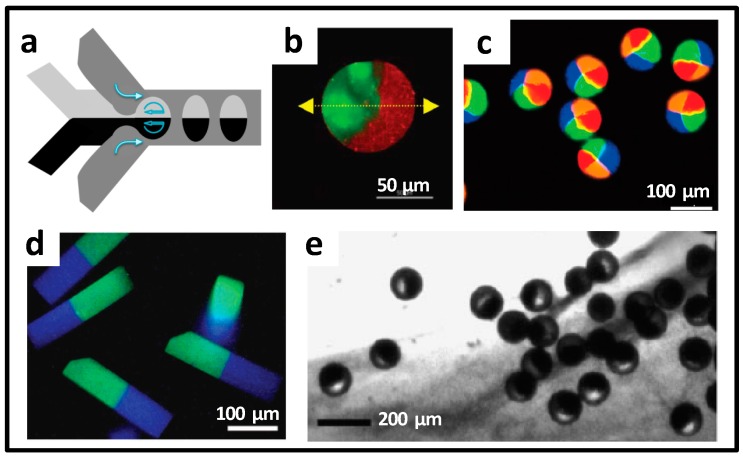
Janus microparticles prepared by microfluidics. (**a**) Microfluidic design for Janus droplet generation; (**b**) Biopolymer-based Janus microbeads with selective degradation; (**c**) Multi-compartment particles obtained from a multi-barreled capillary; (**d**) Janus-type, pH-responsive particles via stop-flow lithography; (**e**) Aggregation and compaction of the Poly(N-isopropylacrylamide) (PNIPAm) microgels. Reproduced with permission from [69,70,71,72].

Janus microparticles are a typical hybrid material. The two parts are different, and thus have multiple properties. In the microchannel, free flowing liquids have laminar properties and can be changed into droplets under the shear stress. With these two features, Janus droplets, as shown in Figure 10a, could be generated, and corresponding Janus microparticles were formed after solidification. Cathala and co-workers induced gelation by diffusion effect and obtained Janus microgels containing two different biopolymers. In particular, biopolymers can be selectively degraded in one hemisphere through enzymatic hydrolysis for the release of a specific inclusion, as shown in Figure 10b [69]. Based on two-phase Janus microparticle synthesis, the researchers further prepared microparticles with three-phase or even multiphase, with axisymmetric structures. Takeuchi et al. arranged the capillaries at a certain angle to control the angular arrangement of different and simultaneous laminar fluids from these capillary arrays. Under the continuous phase shear action offered by the centrifugal force, the multiphase laminar liquid formed multi-component, axial–symmetrical droplets. After the ionic crosslinking reaction, composite microparticles with the calcium alginate as the body, and different hybrid compositions as the compartment, were obtained, as shown in Figure 10c [70]. Cells and magnetic nanoparticles were encapsulated separately in their respective unit areas, which facilitated maintenance of cell viability. Bong et al. utilized microfluidics to synthesize pH-sensitive, multimodulated, anisotropic drug carrier particles, including Janus-type, as shown in Figure 10d [71]. Due to the acidic pH sensibility of the materials, tumor-selective drug release could be realized. The capability of multiple drug encapsulations in the same carrier, and independent release of each drug, rendered the potential of synergistic combinatorial cancer treatment. Besides droplet template method, phase separation effect of droplets can also trigger the formation of the Janus structure. Weitz et al. synthesized Janus microspheres of polyacrylamide and poly (*N*-isopropylacrylamide), as shown in Figure 10e [72].

### 2.2. Particulate Biomaterials at the Nanoscale

Nanoparticles have drawn significant attention, due to their unique and excellent properties. Compared to the batch synthesis process, the microfluidic platform can provide precise control of the temperature, concentration, and mixing process for the synthesis of nanoparticles, thus, the size, size distribution, morphology, reproducibility, and throughput of nanoparticles, could be effectively regulated [73]. The single-phase flow, multiphase flow, multi-step, and coupling system in microfluidic devices are established for the stable microreactor conditions [74]. The synthesis of the particulate biomaterials at the nanoscale include metal nanoparticles, oxide nanoparticles, polymer nanoparticles, hybrid nanoparticles, and quantum dots [75,76], which are applied in bioimaging, biosensing, drug delivery, diagnosis, and therapy. Hassan’s group employed 3D coaxial flow device with a heat supply to fabricate magnetic nanoparticles [77]. Utilizing the multistep microfluidic device with three separated microreactors, fluorescent silica-coated magnetic nanoparticles were synthesized, which could be used for magnetic resonance imaging (MRI) and fluorescence imaging [78]. For molecular imaging, Liu et al. [79] prepared the supramolecular nanoparticles with controllable size and surface chemistry in a polydimethylsiloxane (PDMS) microfluidic chip. Evaluation of cellular uptake efficiency of the nanoparticles displayed the capability in bioimaging. 

**Figure 11:**
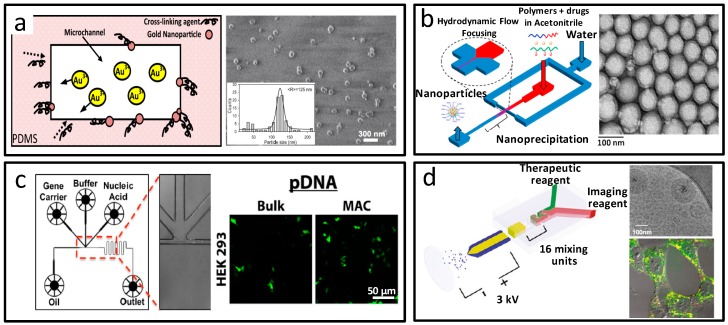
Nanoparticles prepared by microfluidics. (**a**) In situ synthesized gold nanoparticles in the microdevices; (**b**) Dual drug-loaded polymeric nanoparticles by microfluidic self-assembly; (**c**) Microfluidic preparation of polymer-nucleic acid nanocomplexes; (**d**) Theranostic lipoplexes synthesized from microfluidic coaxial electrospray. Reproduced with permission from [82,84,89,94].

Metal nanoparticles used for biosensing, such as silver [80] and gold [81], were first fabricated by microfluidics in single flows, and with more rapid reaction speeds and narrower size distributions compared to those from bulk synthesis. SadAbadi et al. integrated in-situ synthesized gold nanoparticles into microdevices, which served as a localized surface plasmon resonance based biosensor for protein and polypeptide detection, as shown in Figure 11a [82]. The high sensitivity and extreme low detect limits of the detection system suggested clinical application potential. Using segmented flow microfluidic systems, Knauer and co-workers prepared noble metal nanoparticles with core-shell and multi-shell structure [83]. The great change in optical characteristics of these nanoparticles showed promising potential for plasmonic applications.

Biodegradable polymeric nanoparticles with narrow size distribution, high reproducibility, and encapsulation efficiency, are ideal drug and nucleic acid delivery vehicles. Farokhzad’s group prepared dual drug loaded polymeric nanoparticles in the microfluidic device, as shown in Figure 11b [84], by self-assembly of docetaxel, prodrug poly lactic acid (PLA)–Pt(IV), and block polymer polyethylene glycol–poly(lactic-co-glycolic acid) (PEG–PLGA), and subsequent modification of the targeting ligand. Furthermore, they synthesized 45 kinds PEG–PLGA nanoparticles via optimization experiments using multiple precursors [85]. Lipid–polymer nanoparticles for drug delivery included nanoparticles with core–shell structure [86], monolayer or bilayer of lipid shells [87], and hybrid Janus structure [88]. Leong et al. used an emulsion-based microfluidic system to prepare polymer–nucleic acid nanocomplexes with improved nonviral gene transfer capability [89], as shown in Figure 11c. Besides, the small interfering RNA (siRNA) delivery with high loading efficiency and enhanced stability [90], oligodeoxynucleotide delivery with improved cellular uptake and immunostimulatory responses [91], the co-delivery of hydrophilic drug and siRNA [92] with high loading efficiency, and co-delivery of gene and proteins [93], would be achieved by microfluidic fabricated nanoparticles.

For diagnosis and therapy applications, using static micromixer–coaxial electrospray, Lee and co-workers one-step synthesized theranostic lipoplexes, as shown in Figure 11d [94]. The lipoplexes incorporated with QDs, Cy5-labeled antisense oligodeoxynucleotides, as the model imaging reagent, and treatment drug, respectively. Efficient delivery into cancer cells, and down-regulated cancer-related gene expression, revealed the future cancer treatment potential.

It is noted that differences in operating principles of flow-focusing methods do exist among various preparations. For microparticle synthesis, flow-focusing geometries are mainly designed for uniform droplet generation, which are used for microparticle templates. Reaction or changes in the droplets or on the droplet surface induce the generation of the microparticles, while for the nanoparticles, flow-focusing geometries could be used to generate droplet reactors or co-flow reactors [84,95]. For a given set of conditions, such as the channel dimension, channel modification and velocity, and stable co-flows could be generated under flow-focusing designs, as the reactor for nanoparticle synthesis. Among flow-focusing designs for nanoparticle synthesis, 3D hydrodynamic focusing technique requires both horizontal and vertical focusing of the sample flows, and can enhance the uniform mixing by the reduced diffusion length via a microfluidic channel, compared to the 2D hydrodynamic focusing, where the central flow is only focused in the horizontal plane. For example, shape-controlled tetrathiafulvalene–Au hybrid materials, and polyplexes with high uniformity and improved biological performance, could be synthesized by 3D hydrodynamic focusing in straightforward processes [96,97].

## 3. Fibrous Biomaterials Synthesis and Applications

**Figure 12:**
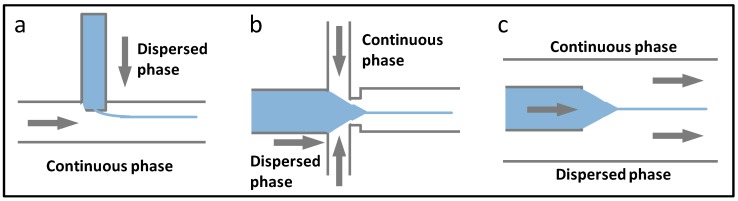
Microfluidic technologies for the synthesis of fibrous materials. The principles and chip designs with different flow regimes for stable co-flow generation, including T-junction (**a**); flow-focusing (**b**); and coaxial (**c**) structured chip.

Microfluidic spinning technology is used as a new technology for synthesizing micro/nano fibrous materials with different shapes, structures, and components, which have been used as scaffolds, with the capability of physiological microenvironment mimicking, cell encapsulation extended to aspects of cell immobilization, co-culture, immunoprotection, and microvascular construction [98]. Except for applications in tissue engineering, controlled drug delivery could also be realized by microfluidic fibrous materials [98]. Microfluidic spinning offers advantages such as the mild fabrication environment, uniformity in the fiber size and shape, and the ability to handle single fibers. Microfluidic microfiber synthesis generally adopts the chip design and manipulation shown in Figure 12, which is analogous to droplet microfluidic chips. That is, by performing the dimension optimization, channel modification, and velocity adjustment to the droplet microfluidic chip, stable co-flows could be generated, which are used as microfiber templates. The principles of fibrous material curing are the same as in Section 2. Based on the above technology and curing principle, microfiber engineered from microfluidics can be divided into solid fiber, special shaped fiber, bamboo structure fiber, core–shell fiber, porous fiber, and multi-component fiber, which are also according to the material shape, structure, and composition. Whilst nanofiber synthesis can be achieved by combining the microfluidic and traditional electrospinning. Besides, special reaction systems are also available.

### 3.1. Fibrous Biomaterials at Micro-Scale

**Figure 13:**
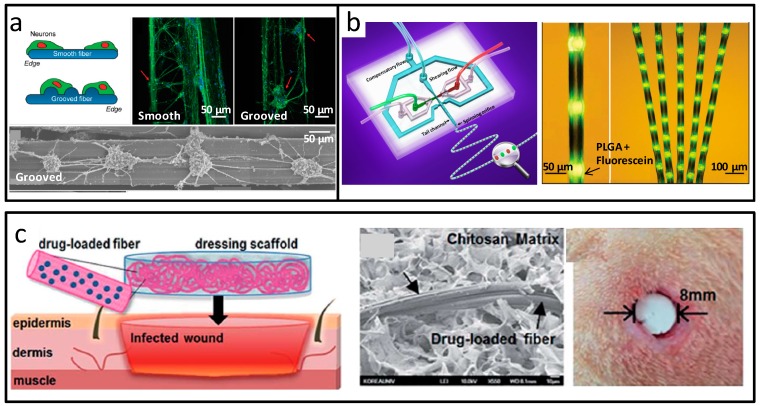
Micro-scale fibers prepared by microfluidics. (**a**) Flat alginate fibers with groove microstructures; (**b**) Biomimetic bamboo-like hybrid microfibers; (**c**) Fibrous alginate carrier from microfluidic spinning. Reproduced with permission from [99,103,105].

Kang and co-workers realized uniform carving microgroove patterns on the surface of flat calcium alginate microfibers by designing a special microstructure in the chip, as indicated in Figure 13a. This structural material can be used as a novel kind of scaffold, to regulate the arrangement of cells in tissue engineering [99]. Hwang et al. synthesized PLGA microfibers with diameters from ten to hundreds of microns in the microfluidic chip. Using different fibers, they found that the cellular orientation could be regulated, which can be applied in microstructure design and construction in regenerative medicine and tissue engineering [100]. Lee et al. developed microfiber spinning for the in situ construction of 3D fibrous scaffolds on the same microfluidic device. Primary hepatocytes were encapsulated, and maintained high viability in the fibrous scaffold over seven days. During the scaffold formation, the soft alginate fibers and encapsulated cells were both without damage [101]. Shi et al. developed a cell-responsive methacrylamide-modified gelatin (GelMA) microfiber based on microfluidic spinning. This fiber possessed microgroove surfaces, and could be used for cell scaffold and cell encapsulation, simultaneously. These microstructured fibers could be used as potential templates for the production of fiber shaped tissues [102]. Yu and co-workers fabricated bamboo like micron-scale bionic material based on droplet microfluidics and wet spinning [103]. As shown in Figure 13b, the fibrous materials were about 100–200 μm in diameter, and can be tens of meters in length. The bamboo pole was natural hydrogel with good biocompatibility, and the bamboo joint was spherical material in an orderly arrangement, which could be microdroplets or multicellular microspheres. The materials could be used as functional carriers of cells and biomolecules. In the work of Kang et al., tunable physiochemical coding systems in the fiber were generated by a novel microfluidic spinning method [104]. The capability of coding diverse materials could be used for the spatiotemporally programmed loading and release of cells or drugs. Ahn et al. developed antibiotic alginate fiber as natural polymer-based drug carriers in the microfluidic spinning system, as indicated in Figure 13c [105]. The materials were with high drug encapsulation efficiency, and a distinctively delayed degradation profile. The densely packed structure and enhanced drug loading efficiency of the fiber were derived from the dehydration effect of polarity isopropyl alcohol in the sheath flow. In vivo infected wound healing showed the application potential. Chu and co-workers reported chitosan microfibers synthesized from microfluidics [106]. The materials were with controllable internal structures, from tubular to peapod-like structures, which could be used for transporting fluid and encapsulation of multiple drugs, respectively.

### 3.2. Fibrous Biomaterials at the Nanoscale

**Figure 14:**
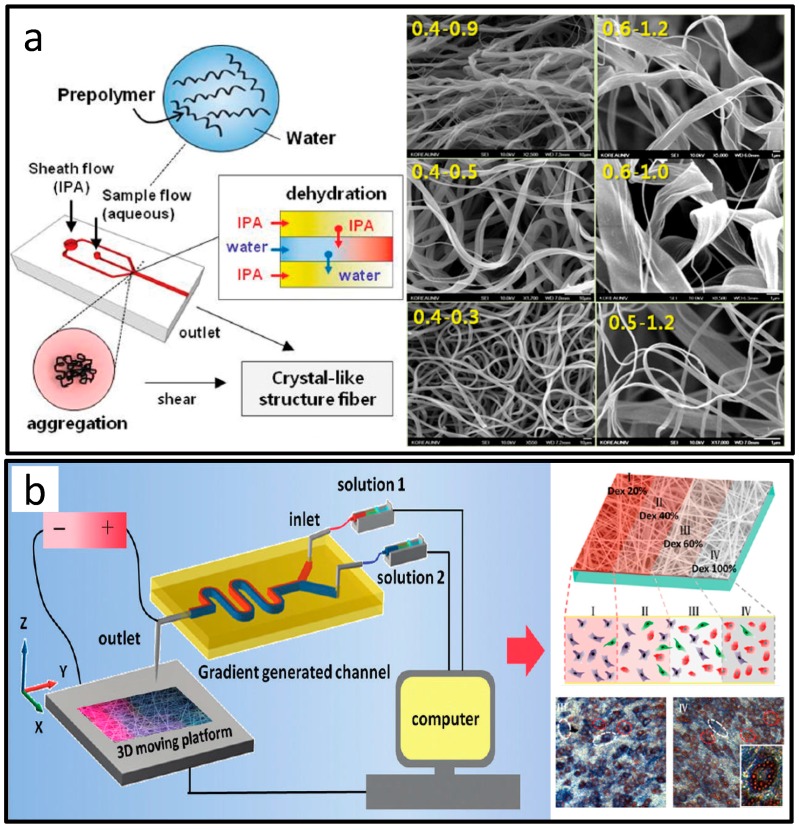
Nano-scale fibers prepared by microfluidics. (**a**) Micro/nanometer-scale fiber with ordered structures; (**b**) Gradient electrospinning nanofibers assisted by microfluidics. Reproduced with permission from [108,109].

The diameters of fibers fabricated by microfluidics range from a few micrometers to hundreds of micrometers [98]. Due to the difficulties in fabricating nanoscale microfluidic channels, fibrous materials scaling down to nanometer are challenging [107]. However, there has still been attempts to synthesize fibrous biomaterials at the nanoscale. Lee et al. prepared nano-scaled alginate fibers using isopropyl alcohol as the sheath flow in the microfluidic system, as shown in Figure 14a [108]. The alginate nanostrands were formed by isopropyl alcohol-induced dehydration. Thinner fibers could be generated from these nanostrands under the shear force in the microchannel into a compact structure. Besides, as shown in Figure 14b, Zhang et al. combined the microfluidic gradient generation and electricspinning, to synthesize the nanofiber scaffold with multiple concentration gradients of large molecular proteins, small molecule drugs, and nanoparticles [109]. The spinning scaffold containing the dexamethasone gradient was used to induce osteogenesis and adipogenic differentiation of mesenchymal stem cells. 

## 4. Sheet Biomaterials Synthesis and Applications

**Figure 15:**
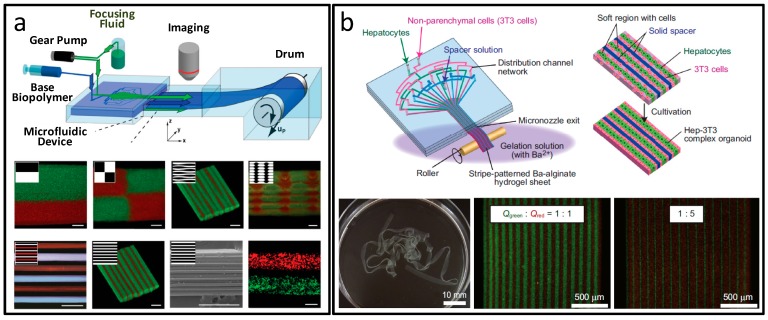
Sheet materials prepared by microfluidics. (**a**) Mosaic hydrogels sheets, scale bars are all 500 μm; (**b**) Micronozzle device patterned hydrogel sheet. Reproduced with permission from [110,112].

Günther et al. developed a digital controlled “textile” technology for mosaic hydrogel sheet synthesis [110]. The highly integrated fluids were regulated through particular programs, and a series of cell patterns were constructed, as indicated in Figure 15a. This work realized high-density cell culture in the unsupported soft material, and offered an idea for research on the relationship between cell–cell, cell–matrix, and 3D functional organization construction in the physiological microenvironment. Hanagata and co-workers in situ synthesized microgroove silica nanotube membranes in a Teflon microfluidic chip [111]. Sustained release of bone morphogenetic protein 2 (BMP-2) and osteoblast differentiation induction could be realized by the membranes, indicating their multiple potentials as cell scaffolds and drug carriers for tissue regeneration. Seki et al. utilized micronozzle device patterned hydrogel sheet for the high-density 3D co-culture of hepatocytes and fibroblasts [112]. As indicated in Figure 15b, HepG2 and 3T3 were co-cultured for seven days in the uniform film of 100 μm in thickness and several millimeters in width, and formed micro-organoids similar to the structure of in vivo hepatic cord. To overcome previous limitations in the scalable formation of polymeric films, such as lack of pore size, shape, and surface control, Elsayed et al. introduced a microfluidic junction and coarse capillaries to synthesize ordered porous films [113]. Highly monodispersed bubbles were firstly generated in T-junction microfluidic capillaries. By regulating the polymer concentration and the surfactant type, microbubbles with controllable pore size were collected on slides to form the porous structures. Selective nanoparticles could be embedded into the films for extensive application in biomedicine.

## 5. Construct Forms of Biomaterials Synthesis and Applications

**Figure 16:**
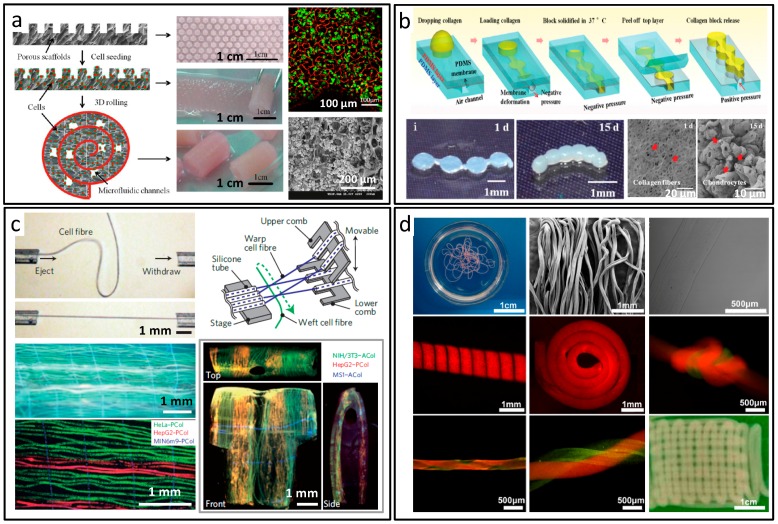
Construct forms of materials prepared by microfluidics. (**a**) Bottom-up fabrication of 3D microfluidic cell-laden constructs; (**b**) Collagen building constructs for self-assembly of 3D microtissues; (**c**) Microfiber-based assembly of 3D macroscopic cellular structures; (**d**) Constructs made by the automated assembly of in situ formed microfibers. Reproduced with permission from [115,116,120,122].

Hydrogel assembly is an effective way to construct soft materials for tissue engineering. Sometimes, due to the poor operation controllability of hydrogels, it remains a challenge to fabricate morphologically accurate structures in vitro. Utilizing microfluidic platforms, assembled microgel material containing different types of cells could be fabricated [114]. As indicated in Figure 16a, in a bottom-up fabrication manner, 3D cell-laden microfluidic constructs were synthesized by Li and co-workers. By rolling monolayer cell-loaded scaffolds, obtained microfluidic channels and primary hepatocytes were uniformly distributed in 3D, which could be potentially used to engineer 3D vascularized liver organs [115]. Qin et al. prepared collagen constructs with tunable geometries through a membrane-templated microdevice, which could be used for 3D construct self-assembly [116], as indicated in Figure 16b. By encapsulating tissue-specific cells into the constructs, functional microtissues were generated. Derived by the combined effects of cell–cell and cell–matrix interactions, cell-laden constructs spontaneous self-assembled into tissue-like constructs. The maximum lengths of tubular constructs, 6 mm, could be achieved by this method. Seki et al. prepared vascular tissues with multilayered, branched, and thick structures, using a microfluidic device made of agarose hydrogel [117]. Smooth muscle cell embedding Ca–alginate hydrogel layer was generated due to the reaction between Ca^2+^ diffused from the agarose channel body and introduced alginate–cell solution. Continuous culture of endothelial cells on this hydrogel layer induced the formation of vascular tissues. More importantly, by removing the agarose hydrogel plates, the vascular tissues could be separated as independent constructs, which enabled vascular tissue based biomedical applications.

There have been several works on the assembly of microfluidic spun microfibers into 3D constructs via reeling, weaving, or direct writing [98]. With accumulated spun microfibers, the dimensions of 3D materials in at least two directions could be at millimeter scale. Fukuda et al. adopted a magnetic-driven strategy to assemble the cell-laden alginate hydrogel microfibers into macroscopic cellular structures with high cell viability [118]. Lee et al. constructed an artificial liver system on the chip by continuously reeling the HepG2-cultured chitosan microfibers [119]. Onoe et al. flexibly handled the cell-laden microfibers using a thin tube and fluid flow, which was revealing for fiber-shaped cellular construct, as shown in Figure 16c [120]. Juncker et al. used a microfluidic direct writer to create 3D cell-laden hydrogel constructs under computer-controlled operating systems [121]. These layer-by-layer assembled 3D structures were with openings permitting media exchange, which was critical for the encapsulated cells. Zhang et al. adopted cell-laden fibers with hierarchically organized architecture for the synthesis of tissue-like constructs, in which the mechanical properties and biological activity could be modulated by the use of photo-cross-linkable methacrylated alginate as the raw materials [122], as shown in Figure 16d.

## 6. Summaries and Outlook

In the last decade, there has been a rapid increase in the number and quality of reports on functional biomaterial synthesis based on microfluidic chip platforms. With the development of microfluidic preparation technology and related detection methods, biomaterials engineered from microfluidic technologies have experienced transition from simplicity to complexity in material structure, and simplification to diversity in material function, including the entire micro/nano-material dimension from 0D particulate materials, 1D fibrous materials, 2D sheet materials, to 3D construct forms of materials, which largely compensates for the lack of conventional synthesis methods. Based on the flexibility and integration of microfluidic manipulation, preparation of functional particulate materials has extended from the original single emulsion droplet method to complex multiple emulsion droplet method, micro-channel limiting, flow lithography, and so on. The resultant particulate materials have developed from simple solid microparticles to specially shaped microparticles, hollow particles, core–shell structured particles, and hybrid particles. A series of novel nanoparticles has also been developed in the microfluidic system. For microfiber materials synthesized from microfluidics, the monotonous structure is gradually changing to complex. More and more works have reported on hybridized microfibers and their functionalization. Meanwhile, the combination of nanospinning technology and microfluidics enables the nanofiber better performance and novel functions. However, there is relatively little work on the microfluidic sheet and construct forms of functional material synthesis, because the increase of material dimension needs complexity enhancement of the microfluidic design, which should be improved by developing more operational microfluidic technology in the future. In stark contrast, microfluidic synthesis of nanoparticles and microfibers are in dynamic development. Table 1 summarizes the dimensions of product, chip design class, materials, and biological applications of biomaterials that are discussed in this review.

**Figure 17:**
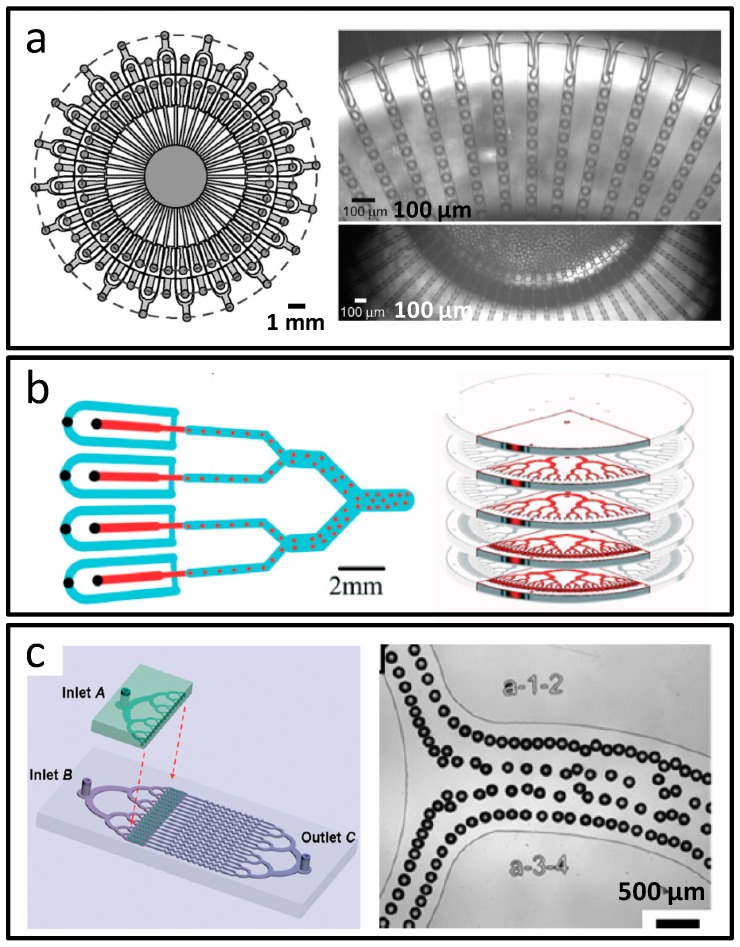
Scaled-up synthesis of functional biomaterials based on microfluidics. (**a**) High-volume production of emulsions in a microfluidic parallelization arrangement; (**b**) A liter per hour volume production of single emulsions in a parallelization chip; (**c**) Particle synthesis in parallel multiple modular microfluidic reactors. Reproduced with permission from [123,124,125].

Based on the development of these microfluidic synthetic technologies, multi-scale structured biomaterials have displayed remarkable properties, including various chemical compositions, high specific surface area and multiscale interface structures, which would be widely applied in the fields of biomedicine, such as drug carrier, cell scaffold, and cellular microenvironment construction. Although microfluidic synthesis has shown significant advantages in both technical and specific applications, as a research field which is at a relatively initial development period, it still faces some pressing problems. Firstly, since current works mostly adopt single-channel as the reaction unit, the yield of microfluidic synthetic materials is low compared to conventional batch mode. To solve this bottleneck, the micro-units in the microfluidic system could be repeated to improve the reaction throughput. Large-scale productions of the microfluidic biomaterials are as shown in Figure 17 [123,124,125], which should be guidance for the industrialization of microfluidic synthesis. The working principles of these three microfluidic modules are based on coaxial annular interfaces (Figure 17a), stacking multiple generator layers (Figure 17b), and parallel multiple modular reactors (Figure 17c), respectively. Next, to highlight the integration strength of the microfluidic platform, automation control should be enhanced to reduce manual operation. Finally, to design and prepare novel biomaterials with more applications, microfluidic platform advantages and practical application of the biomaterials should be dynamically combined. In the future, microfluidic biomaterial design and preparation should be application targeted and oriented.

## Figures and Tables

**Table 1:** Examples of biomaterials synthesized in microfluidic platform.

Dimension of Product	Chip Design	Materials	Potential Biomedical Applications	Reference
0D (spherical microparticles)	“Squeezing out” microchannel	Edible oil	Not mentioned in original work	[37]
0D (spherical microparticles)	Flow-focusing	Poly tripropyleneglycol diacrylate (polyTPGDA) Polyurethane	Not mentioned in original work	[38]
0D (spherical microparticles)	Coaxial	Polyacrylamide (PAM)	Glucose monitoring	[39]
0D (spherical microparticles)	T-junction	Calcium alginate	Cell carrier	[40]
0D (spherical microparticles)	Flow-focusing	Poly(lactide-co-glycolide) (PLGA)	Drug delivery	[41]
0D (spherical microparticles)	Coaxial	Poly(dl-lactic acid) or polycaprolactone (PCL)	Drug delivery	[42]
0D (spherical microparticles)	T-junction variant	Poly(lactic acid) (PLA)	Drug delivery	[43]
0D (spherical microparticles)	Flow-focusing	Silica (SiO_2_)	Sensors Biomolecule delivery	[44]
0D (special-shaped microparticles)	Flow-focusing	PolyTPGDA	Not mentioned in original work	[45]
0D (special-shaped microparticles)	T-junction Flow-focusing	Poly(ethylene glycol) diacrylate (PEGDA)	Drug delivery Biological probes	[46]
0D (special-shaped microparticles)	Coaxial	PLA	Cell trapping or immobilisation	[47]
0D (special-shaped microparticles)	Flow-focusing	SiO_2_	Controlled release Biosensing	[48]
0D (special-shaped microparticles)	Lithography channel	PEGDA	Drug delivery Biosensors	[49]
0D (special-shaped microparticles)	Lithography channel	Colloidal Glass SiO_2_	Biosensor	[50]
0D (core–shell microparticles)	Lithography channel	PEGDA	Cell assembly	[51]
0D (core–shell microparticles)	Flow-focusing Double emulsions	Ferrofluid PAM	Magnetic imaging Micro-mixing	[53]
0D (core–shell microparticles)	Coaxial Double emulsions	PEGDA Ethoxylated trimethylolpropane triacrylate (ETPTA)	Bioassays Cell culture	[54]
0D (core–shell microparticles)	Coaxial Double emulsions	Polyethylene glycol (PEG) Colloidal nanosensors	Biomolecular sensing	[55]
0D (core–shell microparticles)	Flow-focusing	PEGDA	Agent delivery	[56]
0D (core–shell microparticles)	Coaxial	ZIF-8 Alginate	Drug carrier	[57]
0D (core–shell microparticles)	Flow-focusing	SiO_2_	Detoxification	[58]
0D (core–shell microparticles)	T-junction	Poly(styrene-co-acrylic acid)	Ultrasonic MRI	[59]
0D (porous microparticles)	Coaxial	SiO_2_ Silver ETPTA	Molecular detection	[62]
0D (porous microparticles)	Flow-focusing	Poly(GMA-co-EGDMA)	Carriers of biologically active species	[63]
0D (porous microparticles)	Lithography channel	PEG	Biomolecule analysis	[64]
0D (porous microparticles)	Lithography channel	PEG	Protein detection	[65]
0D (porous microparticles)	Flow-focusing	PLGA	Drug carrier Cell carrier	[66]
0D (porous microparticles)	Coaxial	PLGA	Cell scaffold	[67]
0D (composite microparticles)	Flow-focusing	Silver Chitosan	Antibacterial	[68]
0D (composite microparticles)	Flow-focusing	Pectin Alginate Biopolymer	Controlled release	[69]
0D (composite microparticles)	Coaxial Multi-Barrelled Capillary	Calcium alginate	Cell carrier	[70]
0D (composite microparticles)	Lithography channel	PEGDA Ketal-containing diacrylamide	Drug release	[71]
0D (composite microparticles)	Coaxial	PAM Poly(N-isopropylacrylamide) Iron oxide particles	Magnetically manipulation	[72]
0D(nanoparticles)	3D flow-focusing	Goethite	Magnetically manipulation	[77]
0D(nanoparticles)	3D flow-focusing	SiO_2_-coated magnetic nanoparticles	MRI	[78]
0D(nanoparticles)	Digital droplet generator	Supramolecular nanoparticles	Molecular imaging	[79]
0D(nanoparticles)	Single channel	Silver	Biosensing	[80]
0D(nanoparticles)	Single channel	Gold	Biosensing	[81]
0D(nanoparticles)	Single channel	Gold	Biosensor for protein and polypeptide detection	[82]
0D(nanoparticles)	T-junction variant	Au/Ag/Au	Plasmonic application	[83]
0D(nanoparticles)	2D flow-focusing	PEG-PLGA	Drug delivery	[84]
0D(nanoparticles)	3D flow-focusing	PEG-PLGA	Drug delivery	[85]
0D(nanoparticles)	3D flow-focusing	Lipid-PLGA	Drug delivery	[86]
0D(nanoparticles)	3D flow-focusing	Lipid-PLGA	Drug delivery	[87]
0D(nanoparticles)	2D flow-focusing	PEO_45_-b-PS_45_ Platinum nanoparticles Gold nanorods	Drug delivery	[88]
0D(nanoparticles)	T-junction	Polyplexes	Nucleic acid delivery	[89]
0D(nanoparticles)	Y-junction Herringbone structures	Lipid	Nucleic acid delivery	[90]
0D(nanoparticles)	2D flow-focusing	Chitosan	Nucleic acid delivery	[91]
0D(nanoparticles)	3D flow-focusing	Lipid-PLGA	Nucleic acid delivery	[92]
0D(nanoparticles)	Digital droplet generator	Supramolecular nanoparticles	Nucleic acid delivery	[93]
0D(nanoparticles)	Coaxial	Lipoplexes	Cancer treatment potential	[94]
0D(nanoparticles)	3D flow-focusing	Tetrathiafulvalene Au	Not mentioned in original work	[96]
0D(nanoparticles)	3D flow-focusing	Polyplexes	Therapeutics	[97]
1D(microfibers)	Flow-focusing	Calcium alginate	Tissue engineering	[99]
1D(microfibers)	T-junction variant	PLGA	Tissue engineering	[100]
1D(microfibers)	Flow-focusing	Calcium alginate	Tissue engineering	[101]
1D(microfibers)	T-junction	Methacrylamide-modified gelatin or alginate	Tissue engineering	[102]
1D(microfibers)	Flow-focusing	Calcium alginate	Cells or biomolecules carrier	[103]
1D(microfibers)	Flow-focusing	Calcium alginate	Cells or drug delivery	[104]
1D(microfibers)	Flow-focusing	Calcium alginate	Drug carriers	[105]
1D(microfibers)	Coaxial	Chitosan	Drug carriers	[106]
1D(nanofibers)	Flow-focusing	Calcium alginate	Biomimetic material	[108]
1D(nanofibers)	Y-junction	PLGA	Tissue engineering	[109]
2D(sheet)	Multiple parallel microchannels	Calcium alginate	Tissue engineering	[110]
2D(sheet)	Specifical patterning	Silica	Drug delivery Cell guidance	[111]
2D(sheet)	Multiple parallel microchannels	Calcium alginate	Tissue engineering	[112]
2D(sheet)	T-junction	Alginate	Drug delivery Tissue engineering	[113]
3D(constructs)	Specifical patterning	Silk fibroin Chitosan	Tissue engineering	[115]
3D(constructs)	Specifical patterning	Collagen	Tissue engineering	[116]
3D(constructs)	Specifical patterning	Calcium alginate	Tissue engineering	[117]
3D(constructs)	Flow-focusing	Calcium alginate	Tissue engineering	[118]
3D(constructs)	Flow-focusing	Chitosan	Tissue engineering	[119]
3D(constructs)	Coaxial	Calcium alginate	Tissue engineering	[120]
3D(constructs)	T-junction	Calcium alginate	Tissue engineering	[121]
3D(constructs)	Flow-focusing	Calcium alginate	Tissue engineering	[122]
